# Uridine diphosphate-glucuronosyltransferase 2B15 D85Y gene polymorphism is associated with lower prostate cancer risk: a systematic review and meta-analysis

**DOI:** 10.18632/oncotarget.17375

**Published:** 2017-04-24

**Authors:** Xiao Zhong, Jiayu Feng, Ya Xiao, Pingxian Wang, Qiming Fan, Ronghua Wu, Wengang Hu, Chibing Huang

**Affiliations:** ^1^ Department of Urology, Second Affiliated Hospital, Third Military Medical University, Chongqing, 400037, P. R. China

**Keywords:** uridine diphosphate-glucuronosyltransferase 2 family, *UGT2B15*, prostate cancer, single-nucleotide polymorphism

## Abstract

UGT2B15 (uridine diphosphate-glucuronosyltransferase 2B15) catalyzes the conversion of lipophilic C19 steroid androgens such as dihydrotestosterone (DHT) into water-soluble metabolites that can be excreted. Studies of the association between the *UGT2B15* gene D85Y polymorphism and prostate cancer have yielded contradictory results. We therefore systematically searched in the PubMed, EMBASE, Science Direct/Elsevier, CNKI, and Cochrane Library databases, and identified six relevant studies with which to perform a meta-analysis of the relation between *UGT2B15* D85Y polymorphism and prostate cancer risk. Our meta-analysis revealed a significant association between *UGT2B15* D85Y gene polymorphism and prostate cancer in all genetic models (P<0.05). The combined odds ratios and 95% confidence intervals were as follows: additive model, 0.53 and 0.32-0.88; dominant model, 0.51 and 0.33-0.79; recessive model, 0.76 and 0.60-0.96; co-dominant model, 0.55 and 0.35-0.86; and allele model, 0.70 and 0.55-0.89. These results are consistent with the idea that the *UGT2B15* D85Y enzyme variant reduces the risk of prostate cancer by efficiently metabolizing dihydrotestosterone (DHT), which is associated with prostate cancer progression.

## INTRODUCTION

Prostate cancer (PCa) is one of the most frequently diagnosed malignancies in men resulting in more than 250,000 deaths annually [1, 2]. Although PCa is the most common non-cutaneous tumor in developed countries, its etiology remains poorly understood [3, 4]. Identifying risk factors for PCa is critical for developing improved therapeutic interventions and gaining better understanding of the biology of this disease. The major risk factors for prostate cancer include age, race and family history [5–7]. Nearly 42% of the known risk factors of PCa are hereditary and more than 40 single nucleotide polymorphisms (SNPs) are significantly associated with the risk of PCa [8–18].

The enzyme UGT2B15 belongs to a large group of UDP glucuronosyltransferases [19] of the endoplasmic reticulum that inactivates lipophilic C19 steroid androgens, such as dihydrotestosterone (DHT) into water soluble metabolites that can be excreted. *UGT2B15* is expressed in many steroid-sensitive tissues like the prostate [20] and its altered expression is associated with the growth of hormone-refractory PCa [21].

The D85Y polymorphism in the *UGT2B15* gene that results from a single-base-pair change of guanine to thymine leads to a variant with higher V_max_ with reduced risk of PCa [22]. Since UGT2B enzymes are involved in steroid metabolism and excretion, they play an important role in prostate health. However, recent studies that have investigated the association between the uridine diphosphate-glucuronosyltransferase 2B gene polymorphism and prostate cancer have shown contradictory results [23–28]. Some studies reported that the polymorphism increased the risk of prostate cancer, whereas, others did not. Therefore, we systematically reviewed the available literature and performed a meta-analysis to evaluate the association of *UGT2B15* D85Y gene polymorphism with prostate cancer risk.

## RESULTS

### Characteristics of included studies

**Figure 1:**
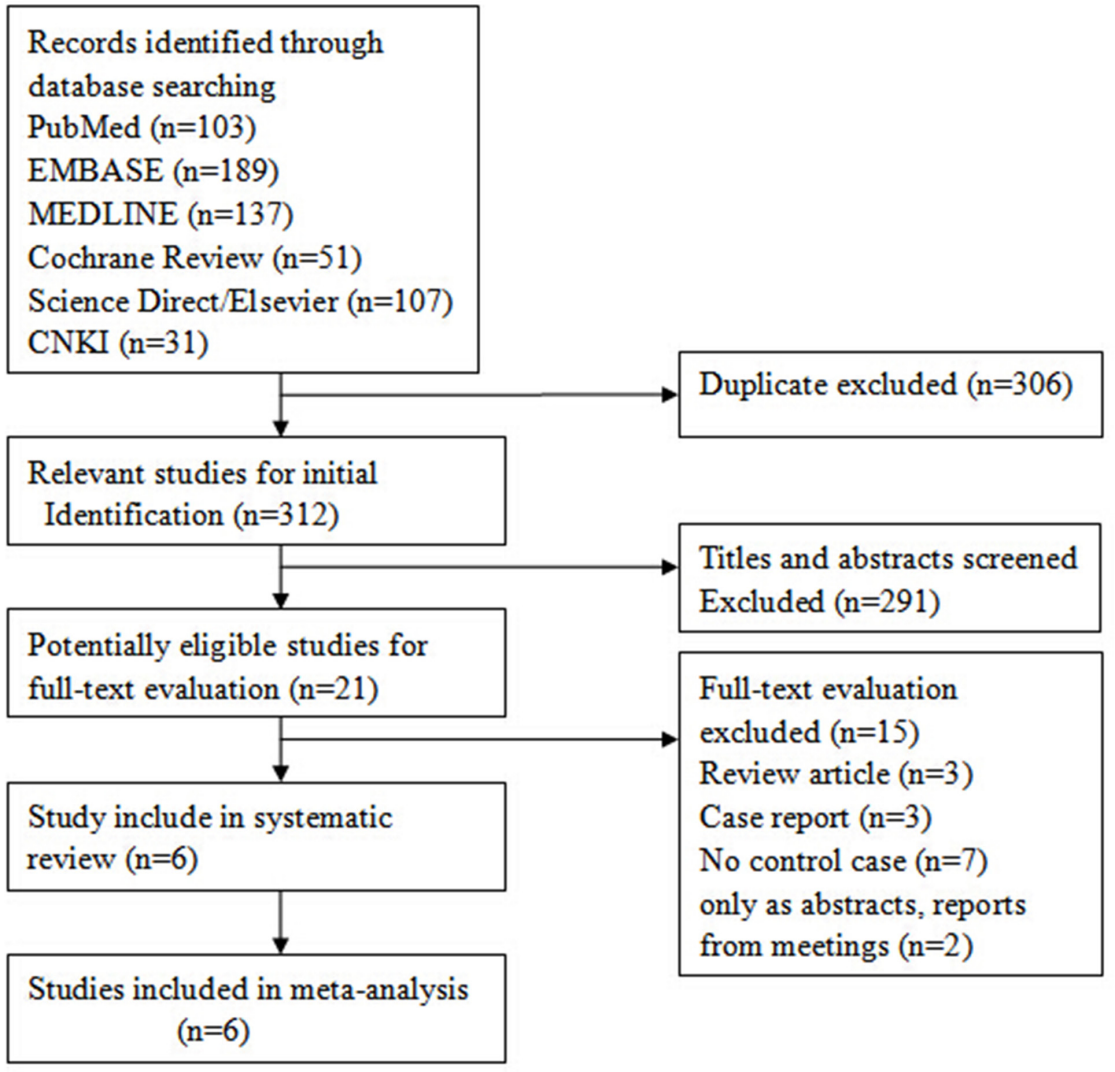
Flow diagram showing selection criteria of eligible studies

The details of the literature review process performed in this study are shown in Figure 1. Initially, a total of 509 unduplicated studies were identified from multiple literature databases and ultimately, six that were in accordance with the eligibility criteria were selected in agreement by all reviewers. The data from the six studies is summarized in Table 1. The six studies included 817 prostate cancer patients and 1000 normal controls and all of them reported exclusion/inclusion criteria [23–28]. In addition, all of these studies tested for the D85Y polymorphism in the *UGT2B15* gene by using restriction fragment length polymorphism (RFLP) analysis after polymerase chain reaction (PCR) amplification.

**Table 1:** Characteristics of the included studies

Author	Country	Case						Control						Genotyping method
		n	D	Y	DD	YD	YY	n	D	Y	DD	YD	YY	
Okugi H 2006	Japan	102	139	65	50	39	13	117	137	97	33	71	13	PCR-RFLP
Gsur A 2002	Australia	190	179	201	40	99	51	190	187	193	47	93	50	PCR-RFLP
Grant D J2013	USA	100	111	73	32	47	13	297	275	319	68	139	90	PCR-RFLP
Park J 2004	USA	155	176	134	52	72	31	154	238	170	21	96	37	PCR-RFLP
MacLeod SL 2007	USA	64	89	39	26	37	1	64	73	55	12	49	3	PCR-RFLP
Hajdinja T 2004	Slovenia	206	195	217	47	101	58	178	148	208	28	92	58	PCR-RFLP

**Abbreviation:** PCR-RFLP: polymerase chain reaction-restriction fragment length polymorphism

### Meta-analysis

**Figure 2:**
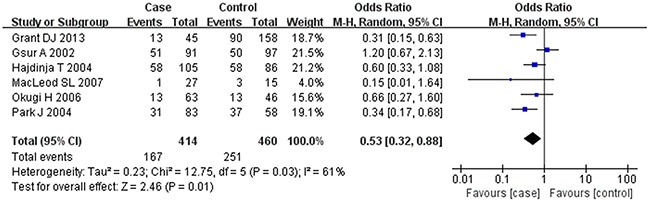
Forest plot showing the meta-analysis outcomes of the additive model

**Figure 3:**
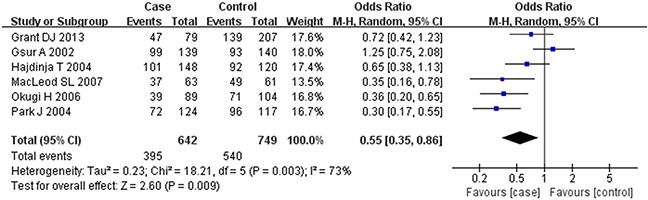
Forest plot showing the meta-analysis outcomes of the co-dominant model

**Figure 4:**
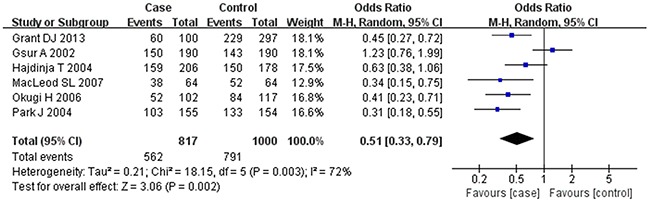
Forest plot showing the meta-analysis outcomes of the dominant model

**Figure 5:**
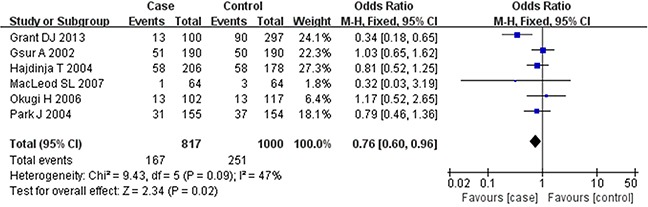
Forest plot showing the meta-analysis outcomes of the recessive model

**Figure 6:**
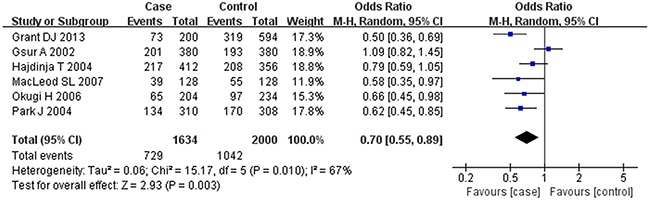
Forest plot showing the meta-analysis outcomes of the allele model

**Figure 7:**
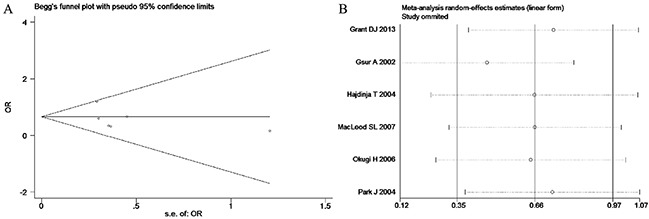
(A) Begg's publication bias and (B) Sensitivity analysis plot of Additive model

**Figure 8:**
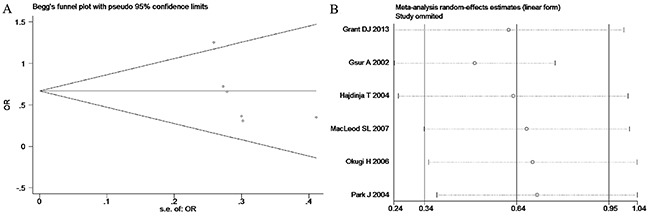
(A) Begg's publication bias and (B) Sensitivity analysis plot of Co-dominant model

**Figure 9:**
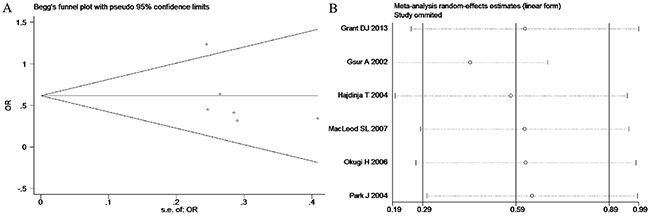
(A) Begg's publication bias and (B) Sensitivity analysis plot of Dominant model

**Figure 10:**
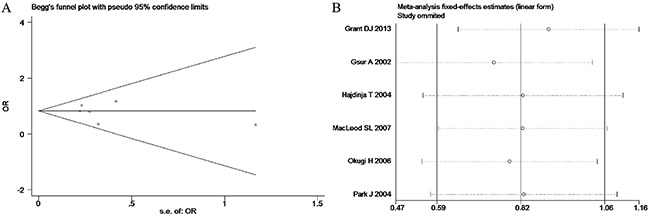
(A) Begg's publication bias and (B) Sensitivity analysis plot of Recessive model

**Figure 11:**
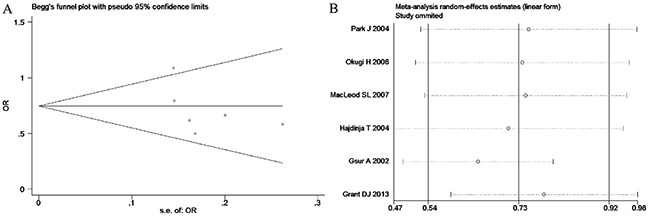
(A) Begg's publication bias and (B) Sensitivity analysis plot of Allele model

Based on the heterogeneity test results, the recessive model was analyzed by the fixed-effects model and the other (additive, dominant, co-dominant and allele) models were analyzed by the random-effects model. The meta-analysis revealed significant association between the *UGT2B15* D85Y gene polymorphism and prostate cancer in all the genetic models (P<0.05). The combined ORs and 95% CIs were as follows: additive model (0.53, 0.32–0.88; Figure 2), co-dominant model (0.55, 0.35–0.86; Figure 3), dominant model (0.51, 0.33–0.79; Figure 4), recessive model (0.76, 0.60–0.96; Figure 5) and allele model (0.70, 0.55–0.89; Figure 6). The symmetric Begg's funnel plots suggested that there was no publication bias in the meta-analysis (Figures 7A, 8A, 9A, 10A, 11A). The Egger's regression test also indicated no evidence of publication bias in all the genetic models (P>0.05; Table 2). Further, we evaluated the sensitivity of the meta-analysis by omitting one study at a time and found that the combined ORs for the remaining studies remained consistent. Overall, no single study significantly changed the combined results indicating that the results were stable and reliable (Figures 7B, 8B, 9B, 10B, 11B).

**Table 2:** Egger's test of publication bias

Models	Coeff.	Std. Err.	t	P>|t|	[95% Conf. Interval]	
Allele	−3.36	2.88	−1.17	0.31	−13.46	5.89
Additive	−1.09	1.36	−0.80	0.47	−4.88	2.69
Dominant	−3.79	3.48	−1.09	0.34	−13.46	5.89
Recessive	−0.51	1.06	−0.48	0.66	−3.45	2.43
Co-dominant	−5.39	3.39	−1.59	0.19	−14.80	4.03

## DISCUSSION

This study focused on six reports in the literature to analyze the association of the uridine diphosphate-glucuronosyltransferase 2B15 gene polymorphism with the risk of prostate cancer. Our results showed that all genetic models (additive, dominant, recessive, co-dominant and allele) demonstrated significant association between the *UGT2B15* D85Y gene polymorphism and prostate cancer. Studies have shown that androgens play important roles in the growth, proliferation and progression of prostate cancer. Therefore, our study showed that the *UGT2B15* gene product that is involved in androgen metabolism played a critical role. Specifically, the data indicated that *UGT2B15* Y85 was protective against prostate cancer.

D85Y polymorphism was non-synonymous with *UGT2B15* gene function [29–31]. Previous studies showed that S-oxazepam was a selective substrate for UGT2B15 and that the common UGT2B15 allozyme variant (D85Y) demonstrated altered S-oxazepam glucuronidation activity [30]. Further, the individual variability of oxazepam glucuronidation by human liver was determined in 35% of cases due to D85Y genotype and 14% of cases due to donor gender [31]. In addition, there was strong correlation between the genetic variants of *UGT2B15* and sipoglitazar exposure with nearly two-thirds of the inter-subject variability in sipoglitazar plasma exposure attributed to the *UGT2B15* genetic variation [32]. All these studies implied that UGT2B15 D85Y was a major determinant of S-oxazepam clearance and a useful *in vivo* probe for glucuronidation by UGT2B15 [33]. Similarly, other studies also demonstrated that the absence of the UGT2B15 protein during early gestation and relatively low expression during late gestation and the neonatal period were consistent with greater risks from some UGT2B15 substrates during these time periods. However, further studies are necessary to confirm these findings.

The UGT (UDP-glucuronosyltransferase) super- family of enzymes comprises endoplasmic reticulum-bound proteins that catalyze the glucuronidation of numerous compounds including drugs, carcinogens and hormones and the resultant inactive polar derivatives are excreted in bile and urine. The UGT1 enzymes on chromosome 2 are primarily involved in the conjugation of bilirubin, phenols, and estrogens. The larger UGT2B enzyme family (on chromosome 4) is grouped into two subfamilies with highly homologous members, namely UGT2A and UGT2B. The *UGT2A* genes are primarily expressed in olfactory epithelium and are involved in terminating odorant signaling. The UGT2B enzymes (UGT2B4, UGT2B7, UGT2B15, UGT2B17, and UGT2B28) are widely expressed in the liver and extra-hepatic steroid target tissues mediating the conjugation of diverse steroid substrates [34, 35]. Sequence divergence in the substrate-binding domains encoded by exons 1 and 2 determines the substrate specificity, whereas the remaining exons encode the conserved UDP-glucuronic acid-binding and the transmembrane domains [36]. Although the association of the UGT2B enzymes with serum steroid levels is unclear, they are important in the biotransformation of steroid hormones and few studies have suggested their association with the risk of prostate cancer [23–28].

As a member of the UGT2B enzyme family, UGT2B15 is a key enzyme for the inactivation of DHT, an active androgen in prostate cells. In the human *UGT2B15* gene, a guanine-to-thymine SNP was identified that resulted in an amino acid change from aspartate (D) to tyrosine (Y) at position 85 [37]. Although both UGT2B15 (D85) and UGT2B15 (Y85) had similar substrate specificities, the V_max_ of UGT2B15 (Y85) was twice that of UGT2B15 (D85) for C19 steroids, such as 5-androstane-3, 17-diol and DHT [38].

Levesque *et al*. reported that the V_max_ of the enzyme resulting from the Y allele was approximately twice compared to the D allele, although no significant differences were evident in the K_max_ of the two enzymes. These findings implied that the D enzyme inefficiently metabolized DHT, resulting in a high DHT concentration in the prostate tissue. Higher levels of DHT were associated with prostate cancer development [39, 40]. Our findings showed association of the D/D genotype with prostate cancer risk supporting the previous findings that associated high DHT with prostate cancer development.

However, there are few limitations in our study that need to be considered when interpreting the results of this meta-analysis. First, the sample size of each of the studies was relatively small with a total of 817 prostate cancer patients and 1000 normal controls investigated in the six studies. Second, several studies had to be excluded from the meta-analysis owing to lack of control data. Because of limited samples, subgroup analysis could not be performed and therefore, definitive conclusions could not be drawn regarding the clinical value of the *UGT2B15* D85Y gene variant in prostate cancer.

In summary, the results of this meta-analysis suggest that the *UGT2B15* D85Y gene variant is protective against prostate cancer. The plausible reason is that the variant has enhanced ability to metabolize DHT, thereby reducing the risk of prostate cancer by decreasing testosterone levels. However, studies with larger sample sizes are necessary to confirm this finding.

## MATERIALS AND METHODS

### Literature search

This meta-analysis was restricted to published studies that investigated the association between *UGT2B15* D85Y gene polymorphism and prostate cancer risk. Two independent reviewers searched PubMed, EMBASE, Science Direct/Elsevier, MEDLINE CNKI, and the Cochrane Library from their inception until June 2016 without restrictions on the language of the report or the study type. The search terms combined text words and MeSH terms. For example, the search terms for the uridine diphosphate-glucuronosyltransferase 2B15 gene were “uridine diphosphate-glucuronosyltransferase 2 family, polypeptide B15” “UGT2B15”, “UDP glucuronosyltransferase 2 family”, “polypeptide B15” or “ugt2b15”; those for prostate cancer were “prostate cancer”, “prostatic neoplasms”, “cancer of prostate”, “cancer of the prostate”, “neoplasms, prostate”, “neoplasms, prostatic”, “prostate neoplasms”, “prostatic cancer” or “PCa”; and those for the polymorphism were “SNP”, “single-nucleotide polymorphism”, “polymorphism”, “variation” or “mutation.” All related articles and abstracts were retrieved. In addition, references cited within relevant reviews were retrieved manually. The search focused only on full articles for the meta-analysis.

### Eligibility criteria

The inclusion criteria for the studies were: (1) Studies that tested the association of uridine diphosphate-glucuronosyltransferase 2B gene variants with prostate cancer; (2) The case groups were prostate cancer patients and healthy individuals were controls and (3) Genotyping for the D85Y SNP was conducted using polymerase chain reaction-restriction fragment length polymorphism (PCR-RFLP). The data extracted from relevant articles included eligible and genotyped cases with controls and the total numbers of cases and controls for each D85Y genotype.

The exclusion criteria were: (1) Studies that were case reports, abstracts, reports from meetings, review articles or duplicated previous publications (2) Studies that lacked a control population and (3) Studies that lacked data on genotype frequencies.

### Study selection and validity assessment

Two independent reviewers screened the titles and abstracts of all citations obtained from the literature search and retrieved those that met the eligibility criteria. If the decision based on the title and abstract was ambiguous, the final decision was made after reviewing the full text. Disagreements were resolved by consensus between the two independent reviewers or decided by a third reviewer.

### Data extraction and statistical analysis

The following data were extracted from the papers by the three reviewers: author names, year of publication, country, the numbers of genotype, genotyping methods, the numbers of cases and controls in each study regarding D85Y genotype and the outcomes. Quantitative meta-analysis was performed and analyzed by the two reviewers using Review Manager (RevMan) software (version 5.2; The Nordic Cochrane Centre, The Cochrane Collaboration, 2012, Copenhagen, Denmark) and Stata software (version 12.0; College Station, TX, USA).

The combined odds ratio (OR) and its 95% confidence interval (CI) were calculated. Heterogeneity or variation between studies was assessed using the P-value and the I-square statistic (I^2^) in the pooled analyses. If the P-value was less than 0.1 or the I^2^-value was greater than 50%, the summary estimate was analyzed in a random-effects model. Otherwise, a fixed-effects model was applied. The association between D85Y polymorphism in the *UGT2B15* gene and prostate cancer risk was investigated in the allelic (Y vs. D), additive (YY vs. DD), dominant (YY and YD vs. DD), recessive (YY vs. YD and DD) and co-dominant (YD vs. DD) models, respectively, where D represents aspartic acid and Y represents tyrosine. Further, publication bias was detected by visual symmetry of the funnel plots, with asymmetry suggesting possible publication bias. It was also assessed by Begg's and Egger's test in the meta-analysis. A P-value of less than 0.05 indicated publication bias.
